# CircSTAM inhibits migration and invasion of trophoblast cells by regulating miR-148a-5p/PTEN axis

**DOI:** 10.1007/s10815-022-02660-4

**Published:** 2022-12-06

**Authors:** Lingfeng Chen, Jinyu Yan, Haiyan Zhang, Jia Xu, Xiaopei Chen

**Affiliations:** Department of Obstetrics and Gynecology, The First People’s Hospital of Wenling, No. 333, Chuan’an South Road, Chengxi Street, Wenling City, 317500 Zhejiang Province China

**Keywords:** CircSTAM, Migration, MiR-148a-5p, PTEN, Preeclampsia

## Abstract

**Background:**

The mechanisms underlying the pathogenesis of preeclampsia (PE) remains unclear. Exploring the molecular players in PE progression can provide insights into targeted therapy.

**Methods:**

The expression levels of circSTAM in placental chorionic tissues of PE patients and normal pregnant women were compared by RT-qPCR. CircSTAM was knocked down by small interfering RNA to investigate its role in migration, invasion and epithelial-mesenchymal transformation (EMT) of trophoblast HTR-8/SVneo cells. The downstream target of circSTAM was predicted using online bioinformatics resources, and their molecular interaction was examined by luciferase reporter assay.

**Results:**

CircSTAM was upregulated in PE placenta tissues in comparison to normal placental tissues. CircSTAM knockdown significantly enhanced cellular invasion, migration, as well as EMT. Mir-148a-5p was identified as a target of circSTAM to regulate cell migration and invasion. Mir-148a-5p negatively regulated PTEN expression in trophoblast HTR-8 /SVneo cells.

**Conclusion:**

In summary, circSTAM upregulation in PE trophoblasts promoted the invasion, migration and EMT. CircSTAM may modulate trophoblast phenotype by impinging on mir-148a-5p/PTEN axis. These data provided novel insights into the pathogenesis of PE.

## Introduction

Preeclampsia (PE), also called prenatal eclampsia, is a pregnancy-associated condition characterized by gestational hypertension, which frequently initiates at 20 weeks of pregnancy [1]. It is recognized as a major cause of mortality and morbidity in pregnant women, with an incidence at 2–8% of all pregnancies worldwide [2, 3]. PE is linked with multi-organ disorder and its pathogenesis involves genetic and environmental factors, such as abnormal immune responses [4], oxidative stress [5], placental deformation and dysfunction, and inflammatory reactions [6, 7]. However, molecular mechanisms underlying the pathogenesis of PE have not been fully understood. Currently, there is no effective treatment strategy for PE, and termination of pregnancy is the only measure for pregnant women with PE diagnosis.

Increasing number of studies have shown the involvement of non-coding (nc) RNAs in the pathogenesis of pregnancy disorder [8–10]. Circular RNAs (circRNAs) are endogenous ncRNAs characterized by covalently linked circular structure and the absence of polyadenylated tail or 5–3 polarity [11, 12]. They are evolutionarily conserved and display tissue-specific expression pattern [13, 14]. CircRNAs are reported to function as miRNA sponge, since they can physically bind to miRNAs and suppress the interaction between miRNAs and corresponding target mRNAs [13, 15]. Several circRNAs have been reported to be potential PE biomarkers [16, 17]. In addition, a recent study has showed that 151 circRNAs were expressed differentially in the blood samples between PE patients and normal controls, and circSTAM was one of them without functional characterization [18]. Understanding potential roles in regulating the trophoblasts (the major players in placental formation) provide more insights into the etiology of PE. We therefore aim to functionally characterize the potential role of circSTAM in regulating the migratory phenotype of trophoblasts.

In the present study, we reported the upregulation of circSTAM in placental chorionic tissues of PE patients, which suggests that circSTAM upregulation may serve as a potential biomarker for PE diagnosis. Moreover, using trophoblast cell line as the in vitro model, we showed that circSTAM targets miR-148a-5p to modulate cellular invasion, migration and EMT of trophoblast cells. We further identified PTEN as a potential target of miR-148a-5p. Together, these findings suggest that circSTAM may contribute to the pathogenesis of PE by regulating the mobility of trophoblasts in the placenta.

## Materials and methods

### Sample collection

The present trial enrolled 45 pregnant females at the First People’s Hospital of Wenling, of whom 25 were diagnosed with PE patients and the other 20 were normal controls (Table 1). All of the subjects were screened strictly for the clinical parameters of PE and for other etiologies [1]. Inclusion criteria: patients with systolic blood pressure ≥ 140 mmHg, diastolic blood pressure ≥ 90 mmHg and urine protein ≥ 0.3 g/24 h. Exclusion criteria: patients with severe PE (systolic blood pressure ≥ 160 mmHg or diastolic blood pressure ≥ 110 mmHg, liver and kidney function damage and visual impairment); patients with eclampsia (convulsions and hyperspasmia that cannot be ascribed to the diagnosis criteria of PE); patients with previous history of frequent abortion; patients with previous history of hypertension.

**Table 1:** The clinical parameters of PE patients and normal controls

Parameters	PE (*n* = 25)	Healthy controls (*n* = 20)	*P* value
Age (years)	28.39 ± 5.13	27.85 ± 4.46	ns
Gestational weeks	32.65 ± 3.60	31.88 ± 3.15	ns
Systolic blood pressure (mmHg)	152.01 ± 6.25	115.68 ± 9.72	***
Diastolic blood pressure (mmHg)	101.44 ± 8.74	72.03 ± 6.96	***
Urine protein (g/24 h)	2.74 ± 1.26	0.16 ± 0.05	**

*ns*, not significant; **P < 0.01; ***P < 0.001

The chorionic tissues of the placenta were collected through abdominal wall puncture or vaginal posterior fornix puncture. The tissues were washed three times with saline and treated with diethyl pyrocarbonate to remove the blood, and then stored in liquid nitrogen instantly until further analysis. Informed consents were acquired from all participants. The experimental procedures were approved by the Medical Research Ethics Committee of the First People’s Hospital of Wenling.

### Quantitative real-time polymerase chain reaction (qRT-PCR)

TRIzol reagent (Invitrogen, CA, USA) was utilized for collecting total RNAs from tissue samples and cell cultures as described in the manufacturer’s instructions. The cDNA synthesis was accomplished with the First Strand cDNA Synthesis Kit (Roche, Swizerland) from RNA sample using random primers. Resulted cDNA was diluted to 40 ng/μL and quantified in a PCR System (BioRad, CA, USA) using SYBR premix EX TAQ II kit (Takara, Dalian, China). The following PCR cycling conditions were used: 95 °C 10 min, 40 cycles of 95 °C 20 s, 58 °C 15 s and 72 °C 45 s. Relative level of target gene expressions was calculated by 2-ΔΔCT approach, with GAPDH and U6 as the internal reference gene. Primers were synthesized by Shanghai Sangon Biotechnology (Shanghai, China):

U6: 5′-CGCAAGGATGACACGCAAAT-3′ (F) and 5′-GTGCAGGGTCCGAG GTATTC-3′ (R); GAPDH: GAPDH, 5′-CCACCCATGGCAAATTCCATGG-3′ (F) and 5′-TCTAGACGGCAGGTCAGGTCCACC-3′ (R); circSTAM: 5′-GAAAG TGATGGAGGCCCTTT-3′ (F) and 5′-TTCAAGATGAAGCAGCTCTGG-3′ (R); mir-148a-5p: 5′-CGGGCAAAGTTCTGTGACACT-3′ (F) and 5′-CAGTGCAGG GTCCGAGGTAT-3′ (R).

### Cell culture and transfection

We procured human extravillous trophoblast HTR-8/SVneo cell line from the American Type Culture Collection (ATCC; VA, USA). Cell cultures were maintained in RPMI-1640 (Gibco) with 10% FBS (Hyclone). Cells were maintained in 6-well plates at 37 °C and 5% CO_2_. CircSTAM small interfering RNA (siRNA) (si-circSTAM) and siRNA negative control (si-NC) were purchased from Syngentech (Shanghai, China). MiR-148a-5p mimic (miR-148a-5p), miR-148a-5p inhibitor and negative controls were produced by Riobo (Guangzhou, China). Transfections of the above molecules were performed by Lipofectamine 3000 (Invitrogen, CA, USA) based on the manufacturer’s protocol. 100 nM of the above molecule was prepared for transfecting cells in 6-well plates at 60% confluency. 48 h after transfection, cells were harvested for further experiments.

### Cell migration and invasion assay

The migratory and invasive capabilities of HTR-8/SVneo cells were assayed by transwell assay. The transwell upper chamber (Corning, Cambridge, USA) was coated with 50 mg/L Matrigel (Gibco, CA, USA, at a 1:8 dilution in PBS) and dried at 37 °C for invasion assay. The upper chamber without Matrigel was used for migration assay. 1 × 10^5^ cells were seeded into the upper chamber in serum-free medium and 1 ml of 10% serum-containing medium was added to the lower chamber. Following incubation (24 h for migration assays; 48 h for invasion assays), the membrane was washed with PBS and cotton tips were used to clean the upper membrane surface. Afterwards, ethanol (95%) was used to fix the lower surface–adherent cells. Fixed cells were stained with 0.25% crystal violet (Sigma, Germany) for 20 min. Cells were imaged under an inverted microscope (Olympus, Tokyo, Japan) at 100 × magnification.

### Wound healing assay

Cell migration was examined by wound healing assay. The cells were inoculated in 6-well plates (1 × 10^6^ cells) until the formation of single cell layer. A scratch was produced in the cell monolayer with a 200-μl-pipette tip. The cells were washed 3 × PBS to remove the floating cells. After re-filling with culture medium, cells were incubated at 37 °C for 48 h. Cell images were captured using an inverted light microscope at 100 × magnifications. The relative migration distance is calculated as ratio of would distance at 48 h/would distance at 0 h.

### RNA pull-down assay

In pull-down experiment, circSTAM probe and the control probe labeled with biotin (Sangon Biotech) were transfected into HTR-8/SVneo cells using Lipofectamine 3000 Reagent (Invitrogen, CA, USA) based on the manufacturer’s protocol. After 48 h, cells were harvested using IP lysis buffer (Beyotime, Beijing, China). The lysate was incubated with streptavidin-coated magnetic beads (MagnaBindTM Streptavidin Beads, Thermo Fisher Scientific, CA, USA) for 4 h to precipitate biotin-coupled RNA complexes. The beads were washed three times with ice-cold lysis buffer. TRIzol reagent (Invitrogen, CA, USA) was utilized to perform RNA purification and the sample was analyzed via qRT-PCR.

### RNase R and actinomycin D treatment

RNase R (Epicentre Technologies, Beijing, China) and actinomyin D (Sigma, Germany) treatment assay was conducted to analyze the stability of circSTAM and GAPDH mRNA. RNA sample was divided equally into two parts: one for Rnase R digestion (Rnase R group), and the other as control group without RNase R treatment. All the samples were incubated at 37 °C for 15 min, and the relative abundance of circSTAM and GAPDH mRNA was examined by RT-qPCR.

RNA stability assay was performed by blocking the transcription using 2 μg/mL actinomycin D. RNA samples were collected at different time points by TRizol reagent. The relative amount of circSTAM and GAPDH mRNA was analyzed by RT-qPCR by normalizing to that before transcription arrest (0 h time point).

### Luciferase reporter assay

Luciferase reporter plasmids with WT or mutated (MUT) binding sites were prepared by Shanghai Sangon Biotechnology (Shanghai, China). 48 h following transfection, cells were lysed and the luciferase activity of the lysate was examined using a Renilla-Firefly Dual Luciferase Assay Kit (Thermo Fisher Scientific, CA, USA). Each sample was normalized by dividing the activity of firefly luciferase with the control renilla luciferase.

### Western blot

Total proteins were extracted using RIPA lysis reagent (Beyotime, Shanghai, China) and the protein contents were measured via BCA kit (Beyotime, Shanghai, China) as per the instructions. Protein samples were separated through SDS-PAGE and transferred to polyvinylidene difluoride (PVDF) membrane (Millipore, MA, USA). After blocking, membranes were labeled with primary antibodies (anti-PTEN, 1:1000, ab245322; N-cadherin, 1:1500, ab19348; E-cadherin, 1:2000, ab133597; GAPDH, 1:2500, ab245355; Vimentin, 1:1000, ab92547; all purchased from Abcam, Cambridge, USA) overnight at 4 °C. Following TBST washing, the membranes were subjected to 2-h incubation with HRP-labeled secondary antibody (1:3000, ab205718) at ambient temperature. Protein band visualization was achieved with an ECL detection kit (Yeasen, Shanghai, China).

### Statistical analysis

Data were expressed as means ± SD (standard deviation) and analyzed by SPSS version 18.0 and Prism GraphPad 8.0 software. Student’s *t*-test was employed to analyze the significance of difference between two groups, and one-way ANOVA and subsequent Tukey’s post hoc test was used for multiple comparisons. All quantification experiments were performed three times and the data were the summary of 3 independent measurements. Differences were regarded as significant when *P* < 0.05.

## Results

### CircSTAM is highly expressed in placental tissues of PE patients

**Fig. 1:**
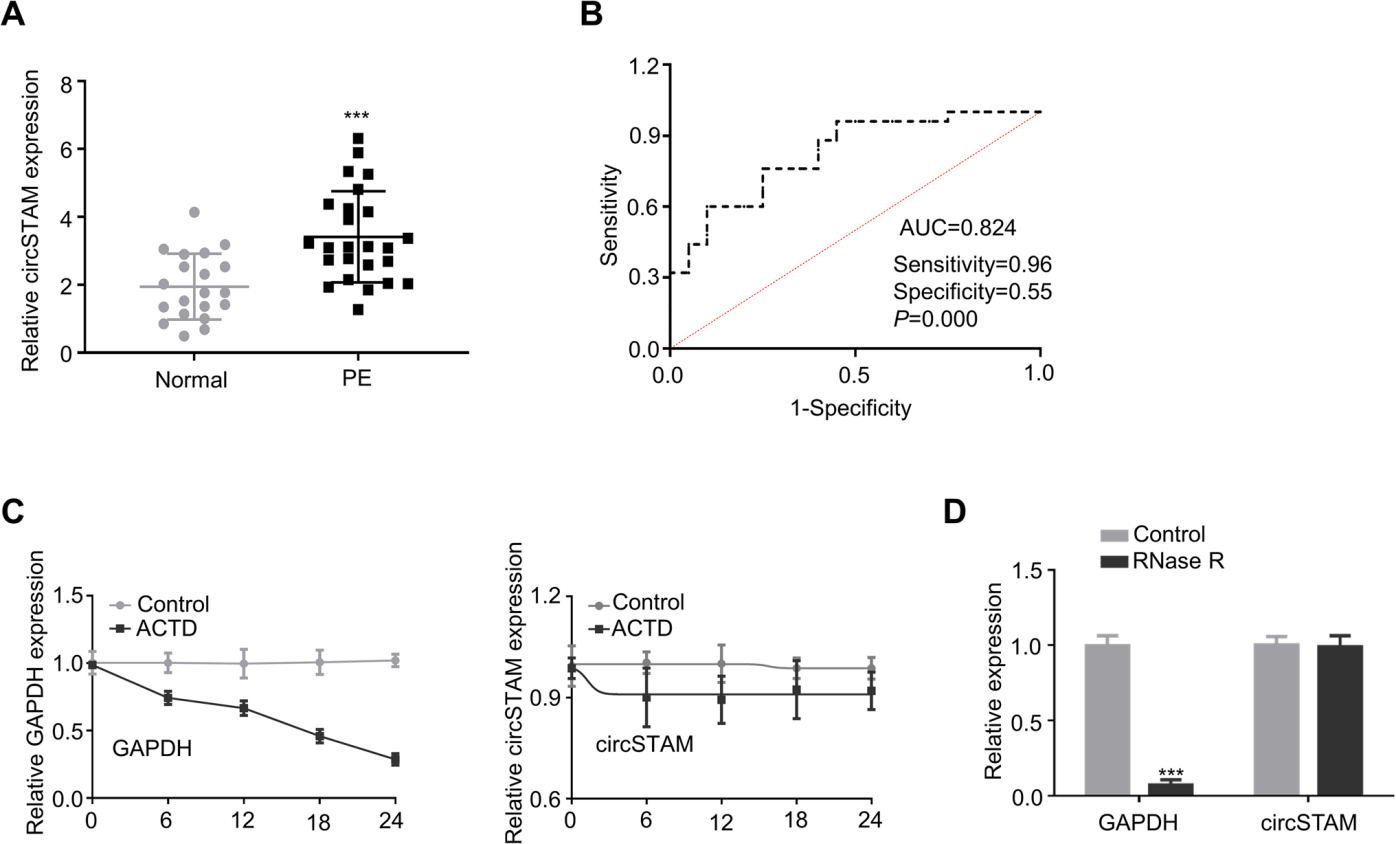
CircSTAM level was high in placental chorionic tissues of PE patients. (**A**) Through qRT-PCR, the placental tissue level of CircSTAM was determined in 20 normal controls and 25 PE patients. (**B**) ROC curve was used to analyze the predictability of circSTAM as a diagnostic indicator in placental tissues of 20 normal controls and 25 PE patients. (**C**) qRT-PCR was performed to determine the expression levels of GAPDH and circSTAM at different time points (0–6-12–18-24 h) following actinomycin D treatment in HTR-8/SVneo cells. (**D**) qRT-PCR was performed to determine the GAPDH and circSTAM expression levels in RNase R–treated or control samples. * *P* < 0.05; **P < 0.01; ***P < 0.001

We first compared the expressions of circSTAM in the chorionic tissues of the placenta between PE patients and normal controls. CircSTAM was highly expressed in placental chorionic tissue of PE patients (Fig. 1A). Receiver operating characteristic (ROC) curve analysis was performed to show the predictability of circSTAM for PE patients. Youden’s index analysis in conjunction with ROC curve suggests that the maximum value of Youden’s index was 0.51, and the AUC (area under the curve) value was 0.824 (*P* = 0.000, sensitivity = 0.96, and specificity = 0.55), suggesting that circSTAM could be used as a potential diagnostic marker for PE (Fig. 1B). To demonstrate the circularity and stability of circSTAM, we performed RNase R and actinomycin D treatment in RNA samples isolated from HTR-8/SVneo cells. As demonstrated by the qRT-PCR findings, GAPDH mRNA level gradually decreased after actinomycin D treatment, while circSTAM level remained relatively stable (Fig. 1C). Additionally, compared with control group, GAPDH mRNA level was reduced after RNase R treatment, while circSTAM level showed no significant change (Fig. 1D).

### CircSTAM knockdown regulates migration, invasion and EMT of HTR-8/SVneo cells

**Fig. 2:**
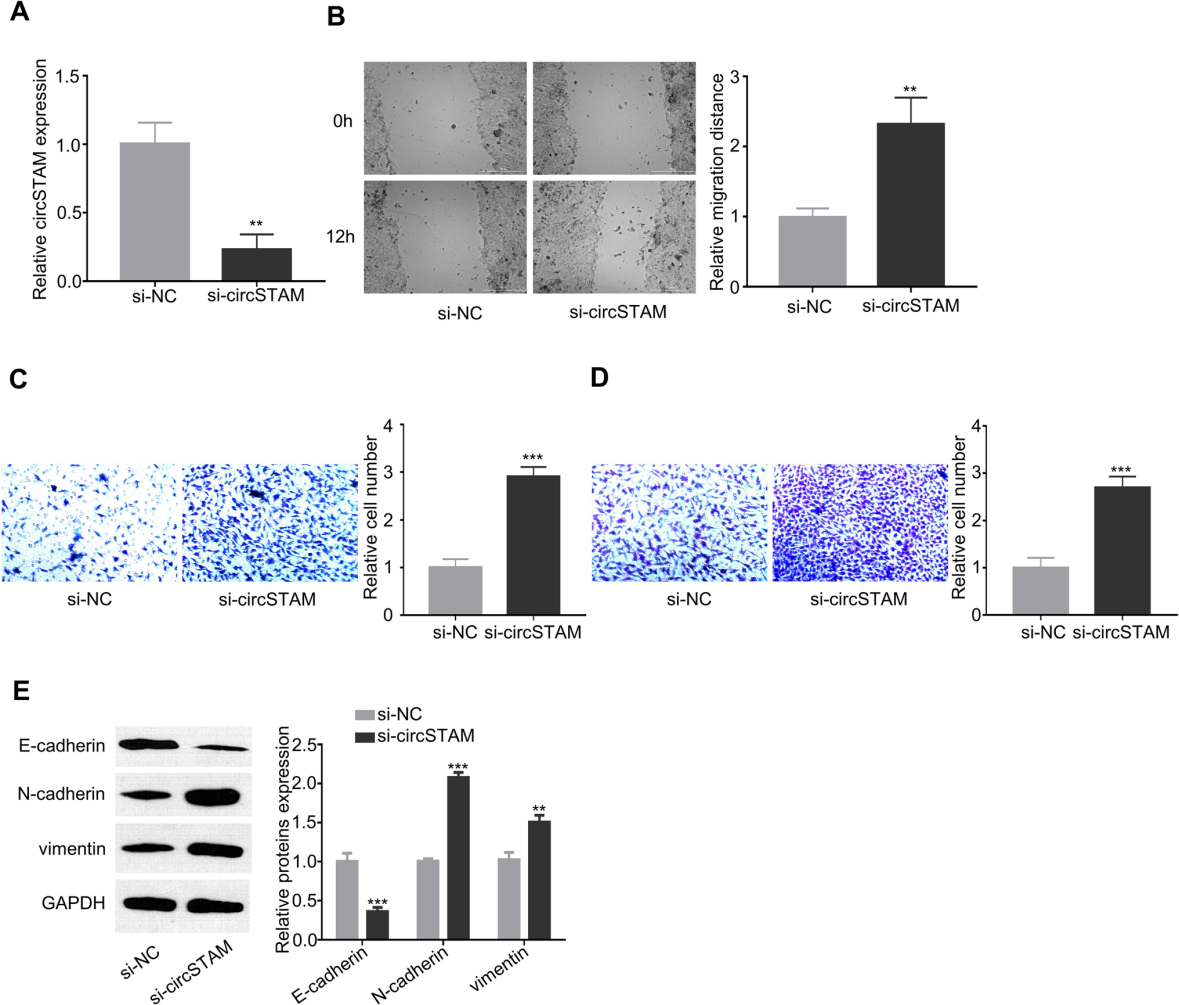
CircSTAM regulates migration, invasion and EMT of HTR-8/SVneo cells. (**A**) CircSTAM expression level in different groups (si-NC and si-circSTAM) of HTR-8/SVneo cells was determined by qRT-PCR. (**B**) HTR-8/SVneo cell migration was assessed by wound healing assay in si-NC and si-circSTAM groups. (**C**) and (**D**) The invasive and migratory capabilities of HTR-8/SVneo cells were assayed by Transwell assay in si-NC and si-circSTAM groups. (**E**) Protein levels of EMT-associated proteins (E-cadherin, N-cadherin and Vimentin) were determined through WB in si-NC and si-circSTAM groups. * *P* < 0.05; **P < 0.01; ***P < 0.001

To clarify the potential roles of circSTAM in trophoblasts, siRNA-mediated gene silencing was conducted to reduce the expression level of circSTAM in HTR-8/SVneo trophoblast cells (Fig. 2A). Upon the silencing of circSTAM, trophoblast cells showed increased migration ability (Fig. 2B). Transwell assay further illustrated that knockdown of circSTAM enhanced the migratory (Fig. 2C) and invasive (Fig. 2D) capabilities. As shown in Fig. 2E, the knockdown of circSTAM caused the increase of N-cadherin and vimentin expression while E-cadherin expression was downregulated, suggesting the role of circSTAM controlling epithelial and mesenchymal transition.

### CircSTAM can target mir-148a-5p

**Fig. 3:**
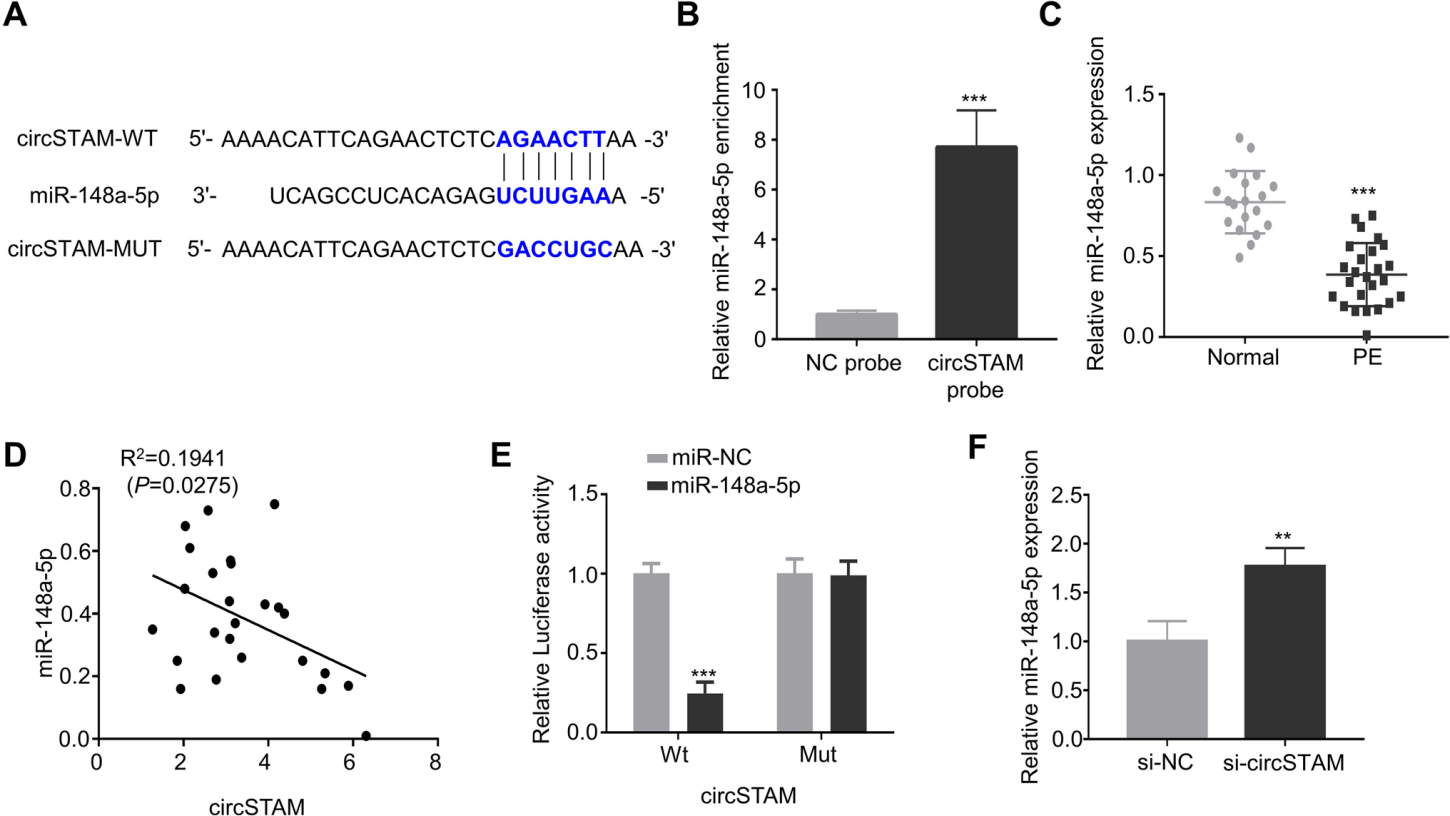
CircSTAM targets mir-148a-5p. (**A**) CircBank predicted the binding sites between circSTAM and mir-148a-5p. (**B**) RNA pull-down analysis using biotin-labeled circSTAM and control probe in HTR-8/SVneo cells. (**C**) Mir-148a-5p was determined based on qRT-PCR in the placental chorionic tissues of 20 normal controls and 25 PE patients. (**D**) The correlation between circSTAM and Mir-148a-5p expression in placental tissues of PE patients. (**E**) Dual luciferase reporter analysis of the interaction between circSTAM and Mir-148a-5p in HTR-8/SVneo cells. (**F**) Mir-148a-5p levels in si-NC and si-circSTAM groups were determined by qRT-PCR. * *P* < 0.05; **P < 0.01; ***P < 0.001

Given the miRNA-sponging activity of circRNA, we further searched the downstream target of circSTAM using circBank prediction, and found that circSTAM contain potential biding sites for mir-148a-5p (Fig. 3A). RNA pull-down analysis using biotin-labeled probe showed that compared with NC probe, circSTAM probe enriched more mir-148a-5p (Fig. 3B). Additionally, the level of mir-148a-5p was significantly lower in placental tissues from PE patients (Fig. 3C). Correlation analysis showed a negative association between circSTAM and mir-148a-5p level in placental chorionic tissues of PE patients (Fig. 3D). Compared with mir-NC, mir-148a-5p mimic could inhibit the luciferase activity of WT luciferase reporter, and the inhibition was abrogated after the predicted circSTAM binding site was mutated (Fig. 3E). Moreover, silencing circSTAM increased mir-148a-5p level (Fig. 3F). Together, these data suggest that circSTAM negatively regulates mir-148a-5p.

### CircSTAM regulates migration, invasion and EMT of HTR-8/SVneo cells through targeting mir-148a-5p

**Fig. 4:**
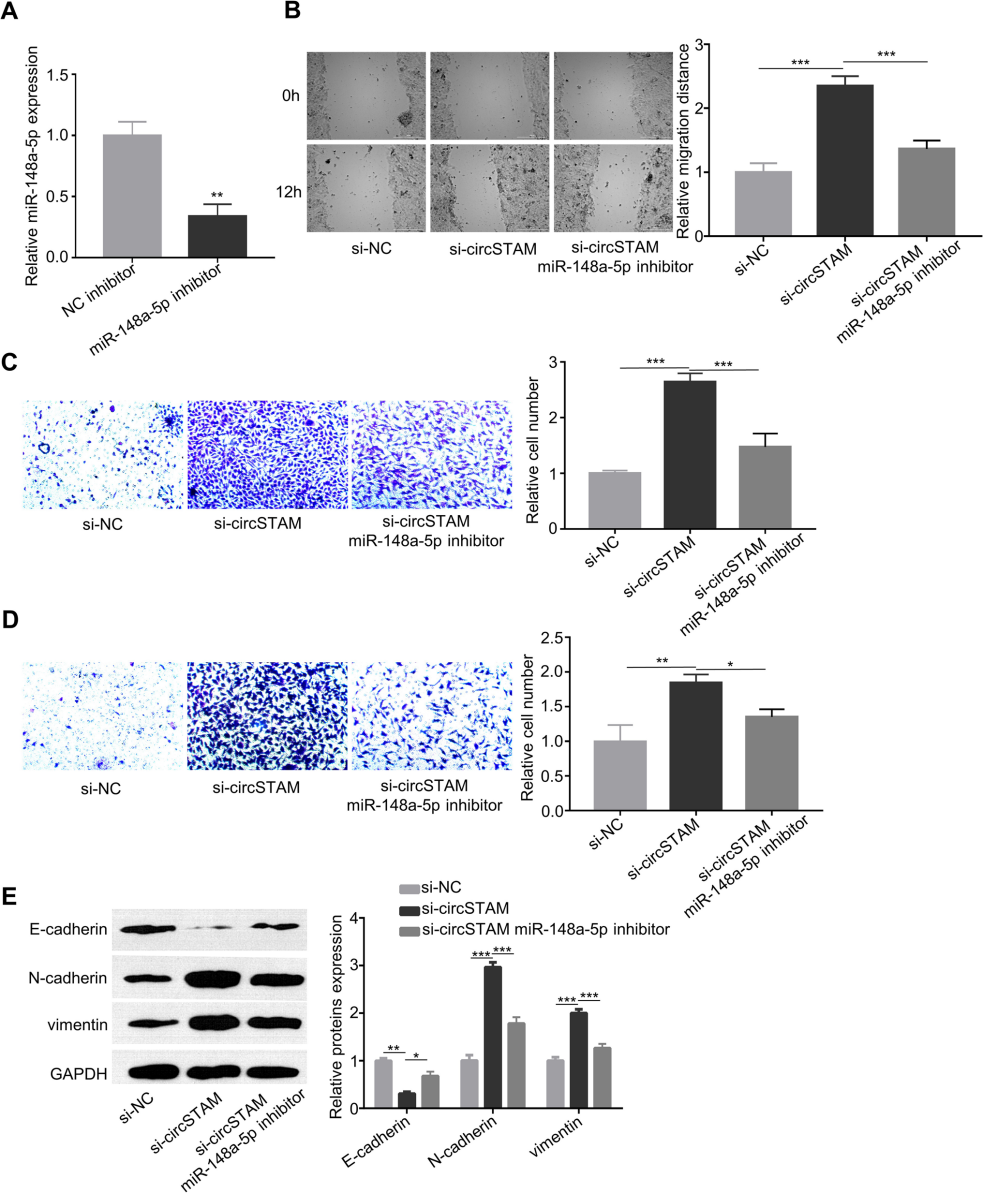
CircSTAM acts on migration, invasion and EMT of HTR-8/SVneo cells through targeted regulation of mir-148a-5p. (**A**) The mir-148a-5p levels in HTR-8/SVneo cells transfected with NC inhibitor and mir-148a-5p inhibitor. (**B**) HTR-8/SVneo cell migration was assessed by wound healing assay in different groups (si-NC and si-circSTam and si-circSTAM + mir-148a-5p inhibitor). (**C**) and (**D**) The invasive and migratory capabilities of HTR-8/SVneo cells were assayed by Transwell assay in different groups (si-NC and si-circSTam and si-circSTAM + mir-148a-5p inhibitor). (**E**) The levels of EMT-associated proteins (E-cadherin, N-cadherin and Vimentin) were determined by WB in various groups of HTR-8/SVneo cells (si-NC and si-circSTAM and si-circSTAM + mir-148a-5p). * *P* < 0.05; **P < 0.01; ***P < 0.001

We further explored whether the interaction between circSTAM and mir-148a-5p regulates the phenotype of HTR-8/SVneo cells. We used mir-148a-5p inhibitor to downregulate cellular expression of mir-148a-5p (Fig. 4A). The co-transfection of mir-148a-5p inhibitor decreased cell migration ability when circSTAM was knocked down (Fig. 4B). In addition, the knockdown of circSTAM increased invasive and migratory capabilities, while the co-transfection of mir-148a-5p inhibitor attenuated these capabilities (Fig. 4C and D). Furthermore, the expression pattern of E-cadherin, Vimentin and N-cadherin induced by circSTAM silencing was also partially reversed by mir-148a-5p inhibitor (Fig. 4E). Therefore, mir-148a-5p is a downstream mediator for circSTAM in trophoblasts.

### CircSTAM sponges mir-148a-5p to regulate the protein level of PTEN

**Fig. 5:**
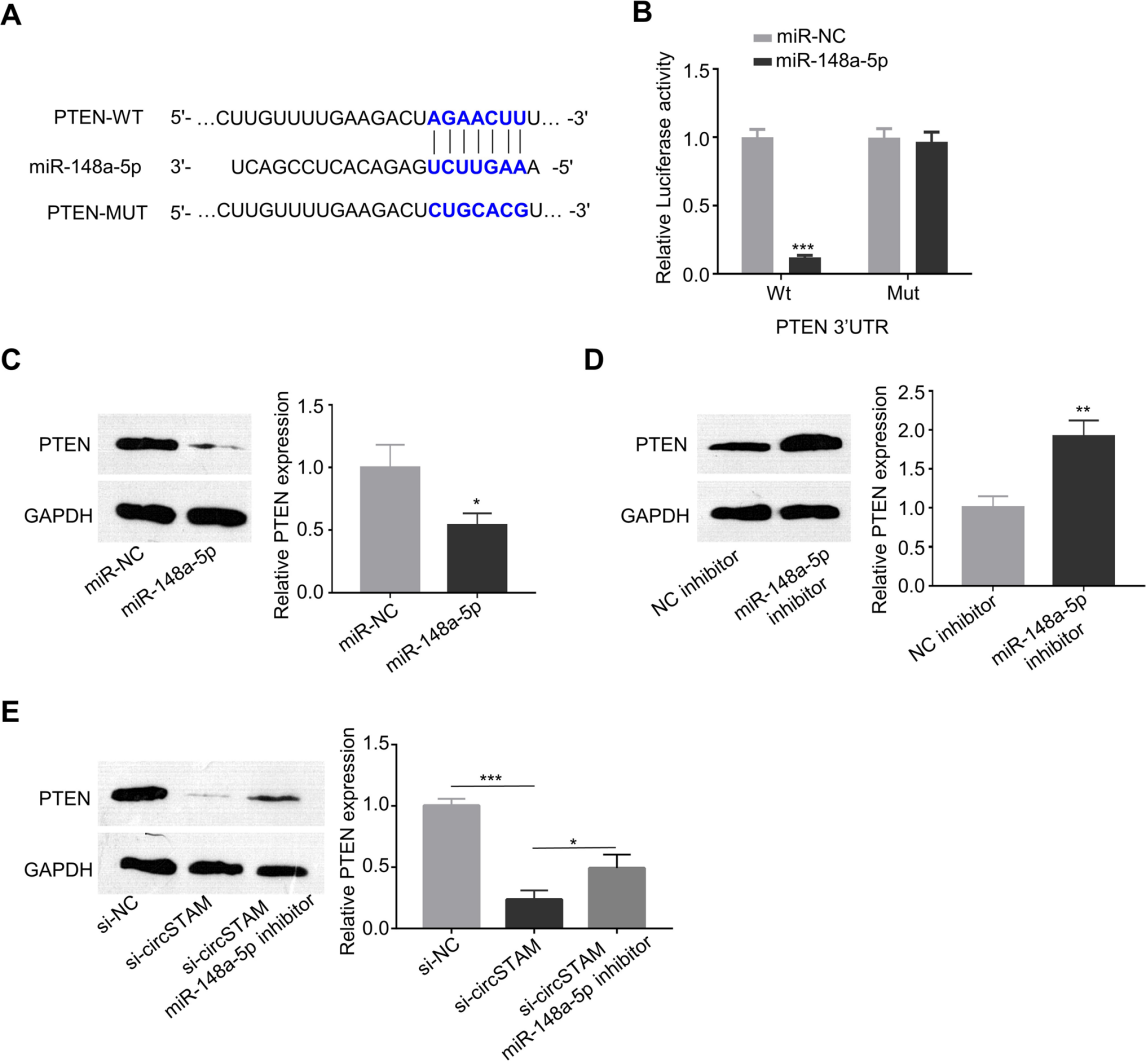
CircSTAM could sponge mir-148a-5p to regulate PTEN expression. (**A**) TargetScan predicted the interaction sites between mir-148a-5p targeting PTEN mRNA. (**B**) Dual luciferase reporter analysis of the interaction between mir-148a-5p and PTEN mRNA. (**C**) PTEN expressions in HTR-8/SVneo cells upon the transfection of mir-NC and mir-148a-5p determined by WB. (**D**) PTEN expressions in HTR-8/SVneo cells upon the transfection of NC inhibitor and mir-148a-5p inhibitor. (**E**) PTEN expressions in various groups of HTR-8/SVneo cells (si-NC and si-circSTam and si-circSTAM + mir-148a-5p inhibitor) were determined through WB. * *P* < 0.05; **P < 0.01; ***P < 0.001

As revealed by TargetScan analysis, there was complementary binding sequences of mir-148a-5p in the 3′ UTR of phosphatase and tensin homolog (PTEN) mRNA (Fig. 5A). To confirm their functional association, we performed dual luciferase reporter assay, in which mir-148a-5p mimic suppressed the luciferase activity of WT reporter while the inhibition was abolished in MUT reporter (Fig. 5B). The transfection of mir-148a-5p mimic significantly reduced the protein level of PTEN (Fig. 5C), and in contrast mir-148a-5p inhibitor transfection elevated the PTEN level (Fig. 5D). Moreover, knockdown of circSTAM reduced PTEN expression, while the co-transfection of mir-148a-5p inhibitor partially increased the protein level of PTEN (Fig. 5E). These data indicate that circSTAM regulates mir-148a-5p/PTEN axis.

## Discussion

CircRNAs have received considerable research attention in cancer biology and in the fields of other pathological conditions. CircRNAs are involved in the regulation of cellular activity mainly through physically adsorbing miRNAs [14]. Their closed-loop structure is resistant to RNase digestion, rendering circRNAs reliable markers for diagnostic purpose [19]. In this study, we showed that circSTAM was highly resistant to RNase R digestion, and was stable after the blockage of transcription, which verified its identity as a circRNA.

Several circRNAs have been found to be differentially expressed in PE patients, which have been proposed as potential markers for diagnosis or targets for treatment [20, 21]. We reported an elevation of circSTAM expression in the placental chorionic tissues of PE patients, as well as a crucial role of circSTAM in regulating the migratory phenotype of HTR-8/SVneo cells. Crosstalk between circRNA and miRNA is a conventional model for competing endogenous RNA regulatory networks. CircRNAs harbor binding sites for miRNAs and are capable of isolating miRNAs from their regulatory function on mRNAs [22]. Our present study showed that circSTAM suppresses the invasion, migration and EMT of HTR-8/SVneo cells by serving as a mir-148a-5p sponge. The proliferation, invasion, differentiation, migration and apoptosis of trophoblast cells are the basic elements of placenta formation and embryonic development [6, 7]. Insufficient trophoblast proliferation is a central cause of PE at early stage. Therefore, our data suggest that circSTAM upregulation in PE patients may impinge on the migratory phenotype trophoblast cells, which may underlie the development of PE.

Mir-148a has previously been reported to be involved in the development of multiple types of cancer, including gastric, hepatic, breast and pulmonary cancers [23]. Furthermore, mir-148a has been described as an integral part of the regulatory circuit of NF-κB pathway, which is activated in Hodgkin and Reed/Sternberg (HRS) cells [24]. However, there is currently no report regarding its role in regulating trophoblast cells. In this study, we found the decreased expression of mir-148a-5p in placental tissues of PE patients. We also reported that mir-148a-5p mediates the role of circSTAM in regulating migration and invasion of HTR-8/SVneo cells. In addition, we also demonstrated that circSTAM could regulate PTEN level by sponging mir-148a-5p. It has been previously reported that PTEN is involved in the molecular pathogenesis of PE development [25]. Specifically, circRNA VRK1 promotes PE progression via sponging miR-221-3P to regulate PTEN/Akt signaling. In addition, long non-coding RNA (lncRNA) LINC01347 also regulates trophoblast migration through the miR-101-3p/PTEN/AKT axis [26]. Therefore, different circRNAs or lncRNAs may connect with different miRNA but regulate similar signaling pathway in PE, such as the regulation of PTEN. On the other hand, it has been reported that metformin shows therapeutic effect for PE by modulating the miR-148a-5p/P28 axis [27]. These findings and our data altogether imply that circRNAs and miRNAs, each can have multiple targets, form an intricate regulatory network in the regulation of PE progression.

Our study is limited by the fact that the cell model is based on the immortalized trophoblast cell line. The observation is required to be validated in the primary trophoblast cells. Furthermore, the mechanisms underlying circSTAM upregulation in PE patients are unclear. Unveiling the molecular mechanism is critical for the formulation of therapeutic strategy to target circSTAM. Further, the role of PTEN dose-dependent effect on the physiological functions of trophoblasts remains to be investigated. Further studies are also needed to consolidate the role of circSTAM in animal model of PE.

## Conclusion

Our study showed an upregulation of circSTAM in placental tissues of PE patients and demonstrated its role in controlling migration and EMT in trophoblasts. In addition, PTEN was identified as target of mir-148a-5p. These data suggest that circSTAM/mir-148a-5p/PTEN axis may underlie the pathogenic development of PE, which requires further validation in animal models.

## Data Availability

The data in the current study are available from the corresponding author on reasonable request.
